# Ischemia-Reperfusion Injury Leads to Distinct Temporal Cardiac Remodeling in Normal versus Diabetic Mice

**DOI:** 10.1371/journal.pone.0030450

**Published:** 2012-02-08

**Authors:** Megumi Eguchi, Young Hwa Kim, Keon Wook Kang, Chi Young Shim, Yangsoo Jang, Thierry Dorval, Kwang Joon Kim, Gary Sweeney

**Affiliations:** 1 Institut Pasteur Korea, Seoul, South Korea; 2 Department of Nuclear Medicine, Seoul National University College of Medicine, Seoul, South Korea; 3 Cardiology Division, Yonsei University College of Medicine, Seoul, South Korea; 4 Division of Endocrinology, Yonsei University College of Medicine, Seoul, South Korea; 5 Department of Biology, York University, Toronto, Canada; The University of Hong Kong, Hong Kong

## Abstract

Diabetes is associated with higher incidence of myocardial infarction (MI) and increased propensity for subsequent events post-MI. Here we conducted a temporal analysis of the influence of diabetes on cardiac dysfunction and remodeling after ischemia reperfusion (IR) injury in mice. Diabetes was induced using streptozotocin and IR performed by ligating the left anterior descending coronary artery for 30 min followed by reperfusion for up to 42 days. We first evaluated changes in cardiac function using echocardiography after 24 hours reperfusion and observed IR injury significantly decreased the systolic function, such as ejection fraction, fractional shortening and end systolic left ventricular volume (LVESV) in both control and diabetic mice. The longitudinal systolic and diastolic strain rate were altered after IR, but there were no significant differences between diabetic mice and controls. However, a reduced ability to metabolize glucose was observed in the diabetic animals as determined by PET-CT scanning using 2-deoxy-2-(^18^F)fluoro-D-glucose. Interestingly, after 24 hours reperfusion diabetic mice showed a reduced infarct size and less apoptosis indicated by TUNEL analysis in heart sections. This may be explained by increased levels of autophagy detected in diabetic mice hearts. Similar increases in IR-induced macrophage infiltration detected by CD68 staining indicated no change in inflammation between control and diabetic mice. Over time, control mice subjected to IR developed mild left ventricular dilation whereas diabetic mice exhibited a decrease in both end diastolic left ventricular volume and LVESV with a decreased intraventricular space and thicker left ventricular wall, indicating concentric hypertrophy. This was associated with marked increases in fibrosis, indicted by Masson trichrome staining, of heart sections in diabetic IR group. In summary, we demonstrate that diabetes principally influences distinct IR-induced chronic changes in cardiac function and remodeling, while a smaller infarct size and elevated levels of autophagy with similar cardiac function are observed in acute phase.

## Introduction

Diabetes is associated with a higher incidence of heart failure and cardiac ischemic events [1]. Patients with both type 1 and type 2 diabetes have a higher risk for encountering sudden death attributed to acute myocardial infarction (MI) and these individuals also exhibit higher mortality rate and risk for developing left ventricular dysfunction after MI [2], [3]. Although the adverse influence of diabetes on left ventricular dysfunction is now a well established phenomenon, the precise temporal nature and mechanisms responsible are still incompletely understood. Due to inherent difficulties in analysis of human myocardium, much of our knowledge on disease mechanisms comes from animal models showing structural, functional or mechanistic characteristics that are observed commonly in diabetic hearts [4], [5], [6]. Use of rodents to examine the influence of diabetes on development and progression of cardiomyopathy has been studied in various transgenic and knockout models in vivo, in isolated perfused hearts and papillary muscles, or in vitro using isolated cardiomyocytes [4], [5]. However, many paradoxical observations exist and there is a need for comparative analysis of remodeling events during the progression of heart failure.

Cardiac remodeling is a progressive process involving changes in hypertrophy, cardiomyocyte apoptosis, inflammation, cardiac metabolism and fibrosis [6]. Each of these is altered in a time-dependent fashion subsequent to ischemia reperfusion injury (IR) that occurs during acute MI. Distinct remodeling events may initially be beneficial as they are initiated to compensate for failing cardiac function but remodeling will ultimately cause transition to heart failure over time [6]. Changes in myocardial metabolism with a reduced glucose uptake and oxidation and thus even higher reliance on fatty acids as a source of energy is one of the first observable remodeling events, often preceding functional indications of a failing heart [4], [6]. Although diabetes has been shown to be associated with increased incidence of acute MI and poorer clinical outcome after IR, the specific effects of diabetes on cardiac function and remodeling, as well as underlying mechanisms, after IR are unclear. Therefore, we designed this study to conduct a time dependent (up to 6 weeks) progressive analysis of IR-induced changes in cardiac performance in control and diabetic mice. We used the streptozotocin-induced diabetic mouse model and induced IR by ligating the left anterior descending coronary artery. The remodeling events underlying changes in cardiac function observed at various times during the course of our study were also investigated.

## Materials and Methods

### Generation of diabetic animals

#### Ethics statement

All animal work must have been conducted according to relevant national and international guidelines. The protocols used were approved by Institut Pasteur Korea Animal Care Committee with reference #IPK11005. Diabetes was induced in male C57BL6 mice (aged 8–10 weeks) via a single dose intraperitoneal injection of 150 mg/kg streptozotocin (Sigma, St. Louis, MO) in 0.1 M citrate buffer, pH 4.1 given one week prior to surgery. Control animals received the citrate buffer for vehicle treatment. In every case we confirmed the blood glucose levels of mice at the time of surgert with the following data obtained: control 143.77±5.89 mg/dL and STZ-treated 501.79±14.06 mg/dL.

### Induction of cardiac ischemia reperfusion injury

The cardiac ischemia reperfusion injury surgery was performed 1 week after the injection of STZ or vehicle solution. The mice were lightly anaesthetized with Zoletil 50 (10 mg/kg) and Rompun (2.5 mg/kg) for intubation with a ventilation tube and they were seduced deeply with an additional 1.5% isofluorane. Left thoracotomy was performed between the fourth and fifth ribs and the pericardial tissue was removed before the ligation of LAD artery. LAD artery was ligated with 7-0 suture around a PF-10 tubing for 30 min and reperfused for 24 hours or 6 weeks. Sham-operated animals underwent the same surgical procedure except that the suture was not tied around the LAD artery. Infarct area was determined by staining the heart sections in 1% 2,3,5-triphenyltetrazolium chloride (TTC, Sigma) solution. Infarct area was calculated as % non-TTC-stained area/total ventricular area.

### Determination of apoptotic cells in cardiac tissue

Apoptotic cells were visualised by TUNEL staining of frozen heart sections using In Situ Cell Death Detection Kit, Fluorescein (Roche) according to the manufacturer's protocol. The quantification of the number of apoptotic cells was performed using an image mining algorithm developed in-house.

### Protein expression determination using Western blotting

Hearts isolated from the animals were snap-frozen and homogenized in homogenization buffer (20 mM Tris-HCl pH 7.5, 150 mM NaCl, 1 mM Na_2_EDTA, 1 mM EGTA, 1% Triton, 2.5 mM sodium pyrophosphate, 1 mM β-glycerophosphate, 1 mM Na_3_VO_4_, 1 ug/ml leupeptin, 1 mM PMSF supplemented with protease inhibitor cocktail (Sigma-Aldrich)). Protein samples (40 ug) were run on SDS-PAGE and transferred onto polyvinylidene fluoride membrane. The membranes were then probed with primary antibodies against LC3B (Cell Signaling), Cathepsin D (H-75), p62 (Sigma-Aldrich), and α-tubulin (P-16, Santa Cruz Biotechnology).

### Echocardiography

Images were obtained using Vevo2100 (Visual Sonics, Toronto, Canada) equipped with MS550D transducer. The mice were lightly anaesthetized using 1.5% isofluorane and restrained on a heated imaging table. The four limbs were attached to the ECG electrodes and hair on the chest was removed using Nair. Images were obtained from the B-mode parasternal long axis view, M-mode of the parasternal short-axis view and the pulse wave Doppler on the mitral valve from the apical four-chamber view to calculate the cardiac diastolic and systolic functions. The echocardiography analysis on animals was performed every week starting 24 hr after the surgery up to 6 weeks. Echocardiograms were stored digitally and strain rate analysis were performed with Vevostrain software (Visual Sonics, Toronto, Canada). All parameters were averaged over at least three cardiac cycles. For quantitative analysis of global left ventricular systolic function, peak systolic strain and systolic strain rate at 6 segments were obtained and the average values were calculated. For quantitative analysis of global diastolic function, peak diastolic strain rate at 6 segments were obtained and the average values were calculated.

### PET imaging and analysis

Whole-body [^18^F]FDG PET/CT was performed using an animal PET/CT scanner (eXplore Vista DR PET/CT, GE Healthcare, Milwaukee, WI). The mice were maintained under fasting condition for 12–14 hours. Thirty minutes before the [^18^F]FDG injection, the animals were treated with oral-administration of glucose (10 mg/g body weight) using zonde to enhance tracer uptake into the myocardium. The PET/CT imaging acquisition was initiated 30 min after the introduction of 18.5 MBq/0.1 mL of [^18^F]FDG via the tail vein injection. Mice were maintained under isoflurane anesthesia (2% in 100% oxygen) on a heating pad during the injection of [^18^F]FDG, uptake period, and PET/CT scanning. Static whole-body PET scans were performed during 10 min in 2 frames. CT scan (40 kV, 250 µA) was obtained during 11.5 min in 2 frames. The images were obtained by Fourier rebinning using ordered subsets expectation maximization (OSEM) reconstruction algorithm with decay-correction, attenuation-correction and random-correction from raw framed sinograms. The data were reconstructed over a 175×175×118 matrix with 0.77-mm slice thickness. Processed images were displayed in coronal, transverse and sagittal planes. For each PET scan, 3-dimensional regions of interest (ROI) were drawn over heart region on whole-body axial images. The standardized uptake value (SUV) for the calibrated and decay corrected series representing 3-D volume data was done as follows: SUV = ROI activity (MBq/g)/[injected dose (MBq)/body weight (g)].

### Histology

The degree of fibrosis was examined by staining frozen heart sections for collagen using Masson Trichrome method. Quantification of the blue area representing collagen was performed as previously described [7] using the Photoshop software. The number of macrophages infiltrating the heart was identified using antibody against CD68 (FA-11, Serotec) and quantified using an in-house image analysis software developed by Dr. Thierry Dorval (Institut Pasteur Korea).

### Statistics

All data are expressed as the means ± SEM. Statistical analysis was performed using t-test or one-way ANOVA and differences were determined to be statistically significant when *P*<0.05.

## Results

### Overall Cardiac function 24 h after IR injury in Diabetic mice

**Figure 1:**
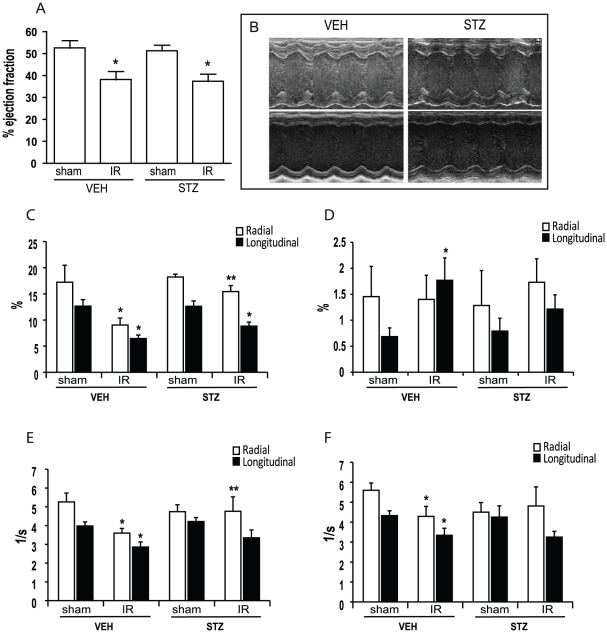
Echocardiography analysis was performed to examine the cardiac function after IR injury. IR injury (30 min) caused a significant decrease in EF in both VEH and STZ animals but no difference was observed between the two IR groups (A). Representative M-mode images from each group where the numbers used for calculations were derived (B). The calculated systolic strain (C), diastolic strain (D), systolic strain rate (E), and diastolic strain rate (F) are derived from cardiac strain analysis. n≥10/group and * indicates that p<0.05 compared to sham of the same treatment group. ** indicates that p<0.05 compared to VEH IR. All values are expressed as absolute values.

Echocardiography analysis at 24 hours reperfusion revealed that IR injury significantly decreased the systolic function in both vehicle treated non-diabetic (VEH) mice and STZ treated diabetic (STZ) mice (table 1 and figure 1). Both ejection fraction (EF) and fractional shortening were significantly reduced in both STZ and VEH animals after IR injury. End systolic left ventricular volume (LVESV) was also increased without any increase in end diastolic left ventricular volume (LVEDV), further indicating a decrease in the left ventricular (LV) contractility. In both groups, the decrease in EF comes from decreased contractility of the anterior wall (figure 1A,B). VEH mice showed a decrease in both radial and longitudinal systolic strain and strain rate whereas STZ mice showed a decrease only in longitudinal systolic strain (table 1, figure 1C&E). VEH mice showed an increase in longitudinal diastolic strain but a decrease in both radial and longitudinal diastolic strain rate, all of which were not affected in STZ mice after IR (figure 1D&E).

**Table 1:** Echocardiographic measurements 24 hours after IR.

	VEH		STZ	
	sham	IR	sham	IR
HR	446.1±15.8	453.7±9.8	397.0±17.8	395.6±19.8
LVEDV (ul)	79.3±3.4	79.8±2.8	72.9±2.6	73.8±2.8
LVESV (ul)	38.1±3.9	52.4±4.1*	35.5±2.4	46.3±3.3*
EF (%)	52.6±3.6	38.2±3.9*	51.3±2.8	37.4±3.4*
FS (%)	27.0±2.3	18.7±2.0*	26.0±1.7	18.1±1.8*
Radial				
Systolic strain (%)	17.2±3.2	9.1±1.3*	18.2±0.6	15.4±1.2**
Diastolic strain (%)	−1.5±0.6	−1.4±0.5	−1.3 0.7	−1.7±0.5
Systolic strain rate (/s)	5.3±0.1	3.6±0.3*	4.7±0.4	5.4±0.5**
Diastolic strain rate (/s)	−5.6±0.4	−4.3±0.5*	−4.5±0.5	−4.8±1.0
Longitudinal				
Systolic strain (%)	−12.7±1.2	−6.5±0.7*	−12.6±1.0	−8.8±0.8*
Diastolic strain (%)	0.7±0.2	1.8±0.4*	0.8±0.2	1.2±0.3
Systolic strain rate (/s)	−4.0±0.2	−2.9±0.3*	−4.2±0.2	−3.7±0.3
Diastolic strain rate (/s)	4.3±0.2	3.3±0.4*	4.3±0.6	3.3±0.3*

*p<0.05 when compared to its own sham.

**p<0.05 when compared to VEH IR.

### Cardiac glucose uptake after IR is reduced in diabetic mice

**Figure 2:**
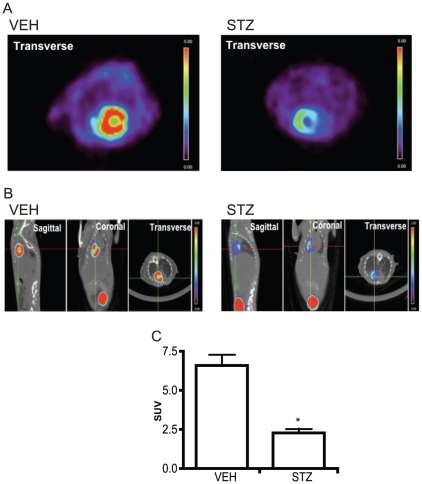
^18^FDG PET-CT was used to examine the level of cardiac glucose uptake in VEH and STZ mice at 24 hr reperfusion. Representative scan images from transverse view (A) and other views (B) from each group are shown. The higher glucose uptake level is shown as the increase in the intensity of red as indicated on the color scale bar shown in each image. Quantification of the accumulated 18FDG (n≥4) in the heart shows that STZ heart has a significantly decreased glucose uptake level (C). * indicates that p<0.05 compared to VEH.

In order to examine the level of cardiac glucose metabolism after IR injury in VEH and STZ mice, ^18^FDG was used to analyze the level of glucose uptake by the heart at 24 hours of reperfusion period. ^18^FDG PET-CT method has been used widely in experimental and clinical settings to quantify cardiac glucose metabolism in the heart [8]. When quantified, total ^18^FDG uptake by the heart was significantly reduced in STZ mice compared to VEH mice (figure 2).

### Infarct size induced by IR is smaller in diabetic mice

**Figure 3:**
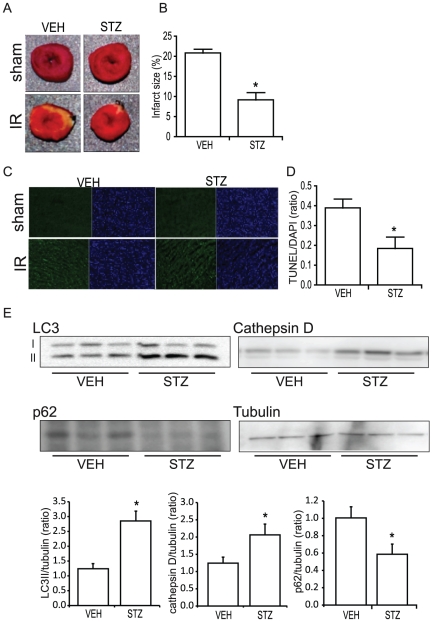
Effects of IR injury on cardiac cell death. A) Hearts were isolated at 24 hr of reperfusion and stained with TTC for the measurement of infarct area. Viable part of the heart appears red and the infarct area white. B) Quantification of the infarct area shows that the infarct area is significantly smaller in STZ compared to VEH hearts. C) Apoptotic cells were identified using TUNEL method. TUNEL-positive nuclei are shown in green and total nuclei in DAPI, blue. D) Quantification of TUNEL positive nuclei reveals that the number of apoptotic cells is much lower in the hearts isolated from STZ animals. Analysis of autophagy-related proteins (E) indicates that autophagy is upregulated in STZ heart. The level of LC3II was significantly higher in the STZ heart compared to VEH and the increase in autophagy was confirmed by the increase in cathepsin D and decrease in p62. (n≥3) and * indicates that p<0.05 compared to VEH.

To examine the effects of STZ-diabetes on the extent of myocardial injury induced by IR, isolated hearts were stained with TTC to identify the infarct area size. Infarct area was significantly reduced in STZ animals compared to VEH animals at 24 hours of reperfusion time as shown visually and quantitatively in figure 3A and B, respectively. We also studied the extent of cell death using TUNEL staining of heart sections prepared after 24 hours of reperfusion. Consistent with infarct area data, the number of apoptotic cells was significantly lower in the STZ mouse heart compared to VEH (figure 3C,D).

### Autophagy is upregulated in diabetic mice hearts

To assess the degree of autophagy in mouse heart, whole tissue homogenate samples were run on Western blot to examine LC3I/II protein level, which is a commonly used marker to determine the extent of autophagy [9]. We observed that STZ-diabetic hearts showed an increased level of LC3II protein (figure 3E), suggesting the upregulation of autophagosome formation. The upregulation of autophagy was further indicated by alterations in expression of cathepsin D and p62 (figure 3E).

### IR-induced macrophage infiltration is not affected by diabetes

**Figure 4:**
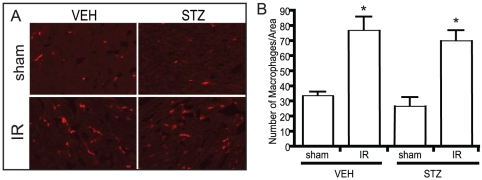
Macrophage infiltration into the heart was examined by immunofluorescent microscopy for macrophage marker CD68. Representative images taken from each group showing the presence of CD68 as red fluorescent (A). Quantification of the number of macrophages in defined area (B). No difference was observed between the VEH and STZ heart at 24 hr of reperfusion. n = 5 and * indicates that p<0.05 compared to sham of the same treatment group.

Macrophage infiltration is part of the local inflammatory response following myocardial infarction and here we examined this process in VEH or STZ mice subjected to IR by staining for the macrophage marker CD68 (figure 4). IR injury increased the number of macrophages recruited into the infarct area to a similar extent in VEH and STZ animals.

### Diabetes leads to distinct progressive changes in cardiac function over time

**Figure 5:**
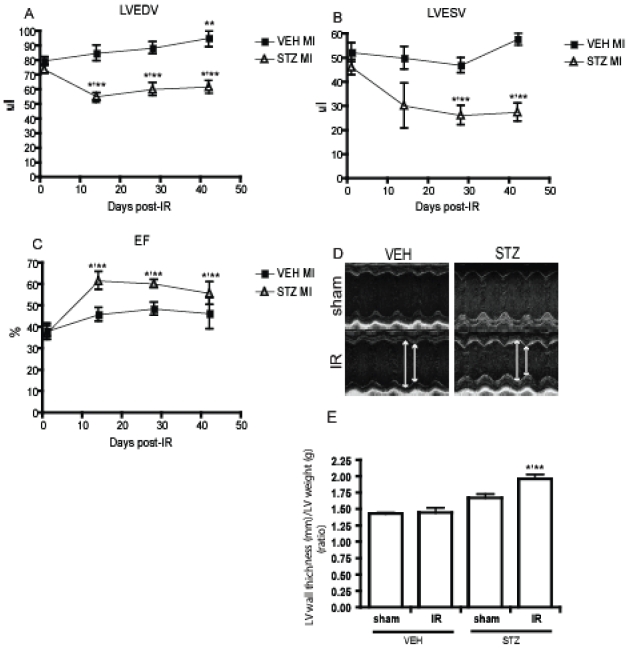
Echocardiography analysis was followed up to 6 weeks of reperfusion to examine the chronic effects of IR injury. VEH mice developed a mild LV dilation as evidenced by a significant increase in LVEDV(A) and a tendency to increase LVESV(B). On the contrary, STZ mice developed concentric hypertrophy as shown by decreased LVEDV, LVESV and increased EF (A,B,C). M-mode image showing the increase in the LV wall and decrease in interstitial space in STZ heart (D). The development of hypertrophy in STZ heart after IR was confirmed by calculating ration of LV wall thickness/LV weight (E). * indicates that p<0.05 compared to Day0 of the same treatment group. **indicates that p<0.05 compared to VEH on the same day post IR and (n≥5).

Over the 6 weeks period that the study was carried out, VEH mice showed a small but significant increase in end diastolic left ventricular volume (LVEDV) (figure 5A). These mice also exhibited a tendency to increase LVESV (figure 5B) but this increase was not statistically significant. STZ animals on the other hand exhibited a decrease in both LVEDV and LVESV. Importantly, diabetic mice also showed an increase in ejection fraction after IR (figure 5C). STZ mice also developed at 42 days a decreased intraventricular space and thicker left ventricular wall suggesting the development of concentric hypertrophy (figure 5D,E).

### Diabetes enhances interstitial collagen deposition after IR injury

**Figure 6:**
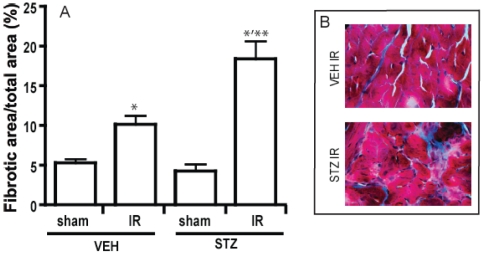
The degree of cardiac fibrosis was accessed by staining collagen using Masson Trichrome method. Quantification of the collagen area over the total area revelaed that IR causes the development of fibrosis in both VEH and STZ heart but the degree of fibrosis is significantly greater in STZ animal (A). Representative image from each group (B). The blue area represents fibrillar collagen. n≥5 and * indicates that p<0.05 compared to sham of the same treatment group. ** indicates that p<0.05 compared to VEH IR.

Fibrosis resulting from collagen deposition was detected using Masson trichrome staining in sections from hearts isolated at 6 weeks post-IR injury (figure 6A,B). Our data revealed an increased level of interstitial collagen deposition in the remote area. STZ-diabetes in sham animals had no effect on the degree of fibrosis, yet the level of collagen deposition was significantly higher in STZ mouse heart after IR compared to VEH heart (figure 6A,B).

## Discussion

Cardiac remodeling is a progressive and dynamic process composed of multiple components that show distinct temporal changes [4], [5], [6]. As mentioned previously, the current literature contains many apparently paradoxical observations on the contribution of diabetes to various remodeling events after the induction of IR. Various explanations are plausible, such as coexistence of obesity, lipotoxicity, activation of renin-angiotensin system and cardiac insulin resistance [4]. Furthermore, we believe one principal factor is that studies were often conducted at a particular time point and although such studies are individually interesting, they neglect the extremely important progressive nature of cardiac remodeling. Here we studied the temporal changes in cardiac structure and function after IR in the non-diabetic and STZ-induced diabetic mouse model and also examined the significance of distinct remodeling events in relation to these changes. The use of the single high-dose STZ injection model was chosen here primarily to address the consequences of hyperglycemia and lack of insulin on cardiac remodeling. It would also be interesting to conduct similar studies in other commonly used obese and diabetic mouse models, in particular high fat diet, although many of these are associated with too many confounding factors in addition to hyperglycemia.

We initially focused on myocardial structural and functional alterations determined using echocardiography. Speckle tracking strain analysis is a novel method which permits the assessment of myocardial deformation in 2 dimensions [10]. In subjects with myocardial infarction, longitudinal strains are significantly reduced proportionately within the area of infarction, and correlate closely with peak infarct mass and ejection fraction. Hearts with smaller infarcts and preserved global left ventricular ejection fraction show sustained radial and longitudinal strain. Therefore, parameters from speckle tracking analysis are regarded as more sensitive than simple conventional echocardiographic parameters to detect myocardial dysfunction [10]. In our study, despite similar reduction of left ventricular ejection fraction after IR, the diabetic group revealed a smaller reduction of radial and longitudianal strain than control group. These findings were consistent with a smaller infarct area in diabetic IR group.

Changes in cardiac metabolism are one of the most important early events in development of cardiomyopathy [4], [6]. Diabetic models consistently demonstrate increased fatty acid utilization and decreased glucose utilization, and the increased use of fatty acid oxidation as a source of ATP generation in the diabetic heart is associated with a higher oxygen consumption which leads to increased oxidative stress and decreased cardiac efficiency [11]. These changes in cardiac metabolism during reperfusion period can have a significant effect on the function of the heart after IR injury [12], [13]. Furthermore, the STZ-induced diabetes model is insulin deficient and likely to exhibit a reduced ability to efficiently use glucose as an energy source. We therefore speculated that the decrease in overall cardiac performance in diabetic animals after IR may be due to reduced ability of the heart to utilize glucose. We observed using [^18^F]-FDG and PET scanning that STZ mice show a significantly decreased level of cardiac glucose uptake compared to non-diabetic mice after IR. With regard to correcting this defect, it has been shown that stimulation of glucose metabolism using pharmacological interventions improves the degree of functional recovery after IR in both animals model and humans [12], [14], [15], [16], [17].

Whereas glucose metabolism decreased, STZ animals exhibited smaller infarct area and fewer TUNEL positive cells after 24 hours of reperfusion. In order to identify a possible mechanism of STZ-diabetes-induced protection against cardiomyocyte injury induced by IR, the level of cardiac autophagy was examined. Autophagy has been observed at elevated levels in the heart of patients or pigs with cardiomyopathy [18], [19], [20] and is thought to play an important role in the regulation of and the development of heart failure [21], [22], [23], [24] and the upregulation of autophagy during ischemia has been shown to be beneficial to the heart [19], [25], [26]. During ischemia, autophagy reduces the level of apoptosis in the heart by degrading proteins and organelles that are damaged and harmful to the cell. The removal of damaged mitochondria is especially important as this will prevent the release of pro-apoptotic factors such as cytochrome c and ROS [23], [27]. Autophagy also enhances the recycling of amino and fatty acids to be used for energy source in conditions of energy shortage such as ischemia [22], [23], [28]. In addition, insulin acting via mTOR is known to inhibit autophagy [29], [30] and in cardiac insulin receptor knockout (CIRKO) mice, there is a constitutively activated level of autophagy [31]. We believe our data indicates that short term activation of autophagy, likely at least in part due to the lack of insulin, in the STZ-diabetic mice may be beneficial in reducing the number of apoptotic cells and infarct size induced by IR. Indeed, Hill's group have shown previously that using aortic constriction to induce pressure overload also induced autophagy in the heart which peaks at 72 hours, is maintained for at least 3 weeks, and provides a protective influence on cardiomyocytes to promote functional recovery [19], [32].

Macrophage infiltration is known to aggravate IR injury [33], [34] and is a potentially important contributor to changes in cardiac function after IR is local inflammation [35]. Macrophages are recruited from circulation to the site of injury and act to initiate the inflammatory response as well as clear debris and contribute to wound healing [35]. Macrophage infiltration into the heart may also be important in effective repair after IR injury as the recruited macrophage participate in producing cytokines and growth factors required for the proliferation of fibroblasts and vascularization [35]. However, long term inflammation is known to induce excessive collagen deposition in the heart and ultimately cause heart failure [36], [37]. Our data indicates that IR induced macrophage recruitment in heart tissue but that the number of infiltrating macrophages was not different between the non-diabetic and diabetic groups after 24 hours of reperfusion.

We performed further echocardiography study on VEH and STZ mice with and without IR over a period of 42 days to obtain a clear understanding of temporal changes in cardiac function. Through this time-course analysis, we observed that VEH and STZ hearts responded differently to IR. IR injury induces a volume overload to the left ventricle and the heart normally compensates for this by mechanically increasing the left ventricle cavity by dilating it [38]. At 42 days post IR, VEH heart showed an increase in LVEDV indicating the development of a mild LV dilation. However on the contrary, STZ animals developed concentric hypertrophy with a decrease in LV volume and intraventricular space and an increase in LV wall thickness. We hypothesized that the STZ heart differently compensate after IR injury by increasing the LV mass as its cavity dilation may have been limited by the excessive accumulation of collagen in the left ventricle of the STZ mice. Indeed, pre-existing fibrosis may be able to prevent dilation of the heart [39] and we observed that IR injury caused a significant increase in the amount of collagen deposition in the remote area, particularly in STZ-diabetic mice. This is also in agreement with established literature indicating that diabetes is associated with enhanced cardiac fibrosis [40], [41], [42]. Our data also indicated that diabetes alone for up to 42 days after sham surgery had no significant effect on the development of fibrosis. Thus, IR injury may have accelerated or enhanced the effects of diabetes on accumulation of collagen in the heart. It is well known that the hemodynamic and neurohormonal changes in the period after MI stimulate events such as intense activation of both the circulating and the local renin-angiotensin-aldosterone system [43]. It has also been reported that diabetes is coupled with an activation of the renin-angiotensin system in the heart [44]. Subsequent reactive and progressive interstitial fibrosis in the heart have been shown to be highly related with elevated antiogensin II and aldosterone. Pathologic changes such as this can induce left ventricular hypertrophy and account for abnormal myocardial stiffness and ventricular dysfunction [45]. Therefore, we suggest that a combination of impaired glucose metabolism and excessive consequences of neurohormonal activation after IR injury would be a possible cause of more severe myocardial fibrosis in diabetic mice.

The higher prevalence of heart failure after MI in diabetic patients [46] can be explained by different chronic cardiac remodeling and fibrosis confirmed in this study. Furthermore, the valsartan in acute myocardial infarction (VALIANT) large scale clinical study of 15,000 patients, used echocardiographic data to clearly demonstrate the existence of the two cardiac hypertrophic responses after IR injury [47], [48]. Concentric hypertrophy was associated with a worse prognosis, higher degree of morbidity and mortality than dilated (eccentric) hypertorophy. Our study also showed that STZ group developed concentric hypertrophy in response to IR injury over time whereas VEH mice responded differently and developed mild LV dilated hypertrophy. Although we did not examine heart failure and mortality at much later time points, the VALIANT findings correlate with the worse prognosis we observed in STZ diabetic mice.

**Figure 7:**
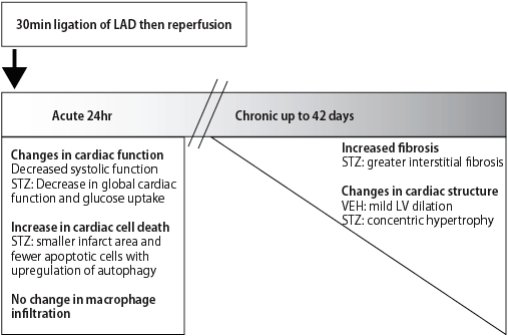
Schematic diagram describing the time span of the experiment with summary of observations made at each time point. Changes in cardiac remodeling events were observed either at acute time point (24 hr reperfusion) or chronic time point (up to 6 weeks reperfusion) after 30 min of ischemia.

In conclusion, we have demonstrated here that STZ-diabetes confers differential effects on cardiac remodeling events after IR injury (figure 7). Diabetic mice show a smaller infarct area post-MI, which may be the result of upregulated autophagy in the heart of these animals. However, in diabetic mice glucose metabolism is compromised at this time. Long term follow up studies indicated that diabetic mice heart develop severe fibrosis and concentric hypertophy leading to detrimental effects on functional performance.

## References

[1] Flaherty JD, Davidson CJ (2005). Diabetes and coronary revascularization.. JAMA.

[2] Fisher BM (1997). Heart abnormalities in IDDM.. Diabetologia.

[3] Katayama T, Nakashima H, Takagi C, Honda Y, Suzuki S (2005). Clinical outcomes and left ventricular function in diabetic patients with acute myocardial infarction treated by primary coronary angioplasty.. Int Heart J.

[4] Bugger H, Abel ED (2009). Rodent models of diabetic cardiomyopathy.. Dis Model Mech.

[5] Breckenridge R (2010). Heart failure and mouse models.. Dis Model Mech.

[6] Abel ED, Litwin SE, Sweeney G (2008). Cardiac remodeling in obesity.. Physiol Rev.

[7] Dahab GM, Kheriza MM, El-Beltagi HM, Fouda AM, El-Din OA (2004). Digital quantification of fibrosis in liver biopsy sections: description of a new method by Photoshop software.. J Gastroenterol Hepatol.

[8] Kofoed KF, Schoder H, Knight RJ, Buxton DB (2000). Glucose metabolism in reperfused myocardium measured by [2-18F] 2-fluorodeoxyglucose and PET.. Cardiovasc Res.

[9] Kabeya Y, Mizushima N, Ueno T, Yamamoto A, Kirisako T (2000). LC3, a mammalian homologue of yeast Apg8p, is localized in autophagosome membranes after processing.. EMBO J.

[10] Geyer H, Caracciolo G, Abe H, Wilansky S, Carerj S (2010). Assessment of myocardial mechanics using speckle tracking echocardiography: fundamentals and clinical applications.. J Am Soc Echocardiogr.

[11] Taha M, Lopaschuk GD (2007). Alterations in energy metabolism in cardiomyopathies.. Ann Med.

[12] Kantor PF, Dyck JR, Lopaschuk GD (1999). Fatty acid oxidation in the reperfused ischemic heart.. Am J Med Sci.

[13] Finegan BA, Lopaschuk GD, Coulson CS, Clanachan AS (1993). Adenosine alters glucose use during ischemia and reperfusion in isolated rat hearts.. Circulation.

[14] Broderick TL, Quinney HA, Barker CC, Lopaschuk GD (1993). Beneficial effect of carnitine on mechanical recovery of rat hearts reperfused after a transient period of global ischemia is accompanied by a stimulation of glucose oxidation.. Circulation.

[15] Lopaschuk GD, Wall SR, Olley PM, Davies NJ (1988). Etomoxir, a carnitine palmitoyltransferase I inhibitor, protects hearts from fatty acid-induced ischemic injury independent of changes in long chain acylcarnitine.. Circ Res.

[16] Lopaschuk GD, Spafford MA, Davies NJ, Wall SR (1990). Glucose and palmitate oxidation in isolated working rat hearts reperfused after a period of transient global ischemia.. Circ Res.

[17] Carvalho G, Pelletier P, Albacker T, Lachapelle K, Joanisse DR (2011). Cardioprotective Effects of Glucose and Insulin Administration While Maintaining Normoglycemia (GIN Therapy) in Patients Undergoing Coronary Artery Bypass Grafting.. J Clin Endocrinol Metab.

[18] Sugimoto S (2007). A novel vacuolar myopathy with dilated cardiomyopathy.. Autophagy.

[19] Yan L, Vatner DE, Kim SJ, Ge H, Masurekar M (2005). Autophagy in chronically ischemic myocardium.. Proc Natl Acad Sci U S A.

[20] Kostin S, Pool L, Elsasser A, Hein S, Drexler HC (2003). Myocytes die by multiple mechanisms in failing human hearts.. Circ Res.

[21] Gottlieb RA, Mentzer RM (2010). Autophagy during cardiac stress: joys and frustrations of autophagy.. Annu Rev Physiol.

[22] Gustafsson AB, Gottlieb RA (2008). Eat your heart out: Role of autophagy in myocardial ischemia/reperfusion.. Autophagy.

[23] Nishida K, Kyoi S, Yamaguchi O, Sadoshima J, Otsu K (2009). The role of autophagy in the heart.. Cell Death Differ.

[24] Whelan RS, Kaplinskiy V, Kitsis RN (2010). Cell death in the pathogenesis of heart disease: mechanisms and significance.. Annu Rev Physiol.

[25] Hamacher-Brady A, Brady NR, Gottlieb RA (2006). Enhancing macroautophagy protects against ischemia/reperfusion injury in cardiac myocytes.. J Biol Chem.

[26] Matsui Y, Takagi H, Qu X, Abdellatif M, Sakoda H (2007). Distinct roles of autophagy in the heart during ischemia and reperfusion: roles of AMP-activated protein kinase and Beclin 1 in mediating autophagy.. Circ Res.

[27] Gustafsson AB, Gottlieb RA (2003). Mechanisms of apoptosis in the heart.. J Clin Immunol.

[28] Rabinowitz JD, White E (2010). Autophagy and metabolism.. Science.

[29] Levine B, Kroemer G (2008). Autophagy in the pathogenesis of disease.. Cell.

[30] Salih DA, Brunet A (2008). FoxO transcription factors in the maintenance of cellular homeostasis during aging.. Curr Opin Cell Biol.

[31] Boudina S, Bugger H, Sena S, O'Neill BT, Zaha VG (2009). Contribution of impaired myocardial insulin signaling to mitochondrial dysfunction and oxidative stress in the heart.. Circulation.

[32] Zhu H, Tannous P, Johnstone JL, Kong Y, Shelton JM (2007). Cardiac autophagy is a maladaptive response to hemodynamic stress.. J Clin Invest.

[33] Lutz J, Thurmel K, Heemann U (2010). Anti-inflammatory treatment strategies for ischemia/reperfusion injury in transplantation.. J Inflamm (Lond).

[34] Sisley AC, Desai T, Harig JM, Gewertz BL (1994). Neutrophil depletion attenuates human intestinal reperfusion injury.. J Surg Res.

[35] Frangogiannis NG, Smith CW, Entman ML (2002). The inflammatory response in myocardial infarction.. Cardiovasc Res.

[36] Haudek SB, Cheng J, Du J, Wang Y, Hermosillo-Rodriguez J (2010). Monocytic fibroblast precursors mediate fibrosis in angiotensin-II-induced cardiac hypertrophy.. J Mol Cell Cardiol.

[37] Ren J, Yang M, Qi G, Zheng J, Jia L (2011). Proinflammatory Protein CARD9 Is Essential for Infiltration of Monocytic Fibroblast Precursors and Cardiac Fibrosis Caused by Angiotensin II Infusion.. Am J Hypertens.

[38] Pfeffer MA (1995). Left ventricular remodeling after acute myocardial infarction.. Annu Rev Med.

[39] Cleland JG, Torabi A, Khan NK (2005). Epidemiology and management of heart failure and left ventricular systolic dysfunction in the aftermath of a myocardial infarction.. Heart.

[40] Aragno M, Mastrocola R, Alloatti G, Vercellinatto I, Bardini P (2008). Oxidative stress triggers cardiac fibrosis in the heart of diabetic rats.. Endocrinology.

[41] Ban CR, Twigg SM (2008). Fibrosis in diabetes complications: pathogenic mechanisms and circulating and urinary markers.. Vasc Health Risk Manag.

[42] Li Q, Wang Y, Sun SZ, Tian YJ, Liu MH (2010). Effects of benazepril on cardiac fibrosis in STZ-induced diabetic rats.. Acta Cardiol.

[43] Foy SG, Crozier IG, Richards AM, Nicholls MG, Turner JG (1995). Neurohormonal changes after acute myocardial infarction. Relationships with haemodynamic indices and effects of ACE inhibition.. Eur Heart J.

[44] Sechi LA, Griffin CA, Schambelan M (1994). The cardiac renin-angiotensin system in STZ-induced diabetes.. Diabetes.

[45] Weber KT, Brilla CG (1991). Pathological hypertrophy and cardiac interstitium. Fibrosis and renin-angiotensin-aldosterone system.. Circulation.

[46] Aneja A, Tang WH, Bansilal S, Garcia MJ, Farkouh ME (2008). Diabetic cardiomyopathy: insights into pathogenesis, diagnostic challenges, and therapeutic options.. Am J Med.

[47] Verma A, Anavekar NS, Meris A, Thune JJ, Arnold JM (2007). The relationship between renal function and cardiac structure, function, and prognosis after myocardial infarction: the VALIANT Echo Study.. J Am Coll Cardiol.

[48] Verma A, Meris A, Skali H, Ghali JK, Arnold JM (2008). Prognostic implications of left ventricular mass and geometry following myocardial infarction: the VALIANT (VALsartan In Acute myocardial iNfarcTion) Echocardiographic Study.. JACC Cardiovasc Imaging.

